# Investigation of the functional role of human Interleukin-8 gene haplotypes by CRISPR/Cas9 mediated genome editing

**DOI:** 10.1038/srep31180

**Published:** 2016-08-08

**Authors:** Manjunatha R. Benakanakere, Livia S. Finoti, Urara Tanaka, Gregory R. Grant, Raquel M. Scarel-Caminaga, Denis F. Kinane

**Affiliations:** 1 Department of Periodontics, School of Dental Medicine, University of Pennsylvania, Philadelphia, PA, USA; 2 Department of Oral Diagnosis and Surgery, School of Dentistry at Araraquara, UNESP- Univ Estadual Paulista, SP, Brazil; 3 Department of Genetics, Perelman School of Medicine, University of Pennsylvania, PA, USA; 4 Department of Morphology, School of Dentistry at Araraquara, UNESP- Univ Estadual Paulista, SP, Brazil; 5 Department of Pathology, School of Dental Medicine, University of Pennsylvania, Philadelphia, PA, USA

## Abstract

Interleukin-8 (*IL-8*) gene polymorphisms have been considered as susceptibility factors in periodontal disease. However, the functional roles of *IL-8* gene haplotypes have not been investigated. Here, we demonstrate for the first time the use of the CRISPR/Cas9 system to engineer the *IL-8* gene, and tested the functionality of different haplotypes. Two sgRNAs vectors targeting the *IL-8* gene and the naked homologous repair DNA carrying different haplotypes were used to successfully generate HEK293T cells carrying the AT genotype at the first SNP - rs4073 (alias -251), TT genotype at the second SNP - rs2227307 (alias +396), TC or CC genotypes at the third SNP - rs2227306 (alias +781) at the *IL-8* locus. When stimulated with Poly I:C, ATC/TTC haplotype, cells significantly up-regulated the *IL-8* at both transcriptional and translational levels. To test whether ATC/TTC haplotype is functional, we used a trans-well assay to measure the transmigration of primary neutrophils incubated with supernatants from the Poly I:C stimulation experiment. ATC/TTC haplotype cells significantly increased transmigration of neutrophils confirming the functional role for this *IL-8* haplotype. Taken together, our data provides evidence that carriage of the ATC/TTC haplotype in itself may increase the influx of neutrophils in inflammatory lesions and influence disease susceptibility.

Interleukin-8 (*IL-8*) is a pro-inflammatory chemokine produced by cells such as epithelial cells, fibroblasts, endothelial cells, macrophages, lymphocytes and mast cells upon exposure to the inflammatory milieu^1^. *IL-8* secretion leads to activation and migration of neutrophils from the peripheral blood to sites of infection that manifest in the clearance of pathogens. Controlled induction of *IL-8* is crucial in the maintenance of homeostatic balance. For example, elevated *IL-8* induction can lead to exacerbated inflammation in chronic inflammatory diseases^2^,^3^. On the contrary, inhibition of *IL-8* secretion may delay neutrophil influx creating an advantage for pathogen survival leading to chronic infection.

Elevated *IL-8* expression has been attributed to a number of diseases such as chronic obstructive pulmonary disease^4^,^5^, hypertension^6^, carcinogenesis^7^,^8^, idiopathic pulmonary fibrosis^9^,^10^,^11^ and chronic periodontitis^12^. Previous studies have investigated the association of single nucleotide polymorphisms (SNPs) with the level of *IL-8* gene expression. The SNP rs4073 (alias -251) in the *IL-8* gene has been considered functional, since the -251A allele was related to higher levels of IL-8 production *in vitro*, after stimulation with lipopolysaccharide and cytokines^13^. This agrees with the finding that the AA genotype of -251 SNP in the *IL-8* gene was associated with greater *IL-8* mRNA expression^14^. However, another study demonstrated that the TA genotype in this -251 SNP was associated with increased *IL-8* mRNA levels^15^.

The genetic polymorphisms are prevalent in any given population and are often reported to differ between health and disease^16^,^17^. The association between SNPs and periodontitis is well documented^17^,^18^,^19^. In particular, −251(T/A), +396(T/G) and +781(C/T) polymorphisms in the *IL-8* gene, forming the haplotype ATC/TTC is associated with periodontal disease and carriers of this haplotype had 2 times higher disease susceptibility than the other haplotypes, such as the ATT/TTC, which was not associated with susceptibility to periodontal disease^20^. Numerous reports investigated whether polymorphisms in this gene influence the levels of periodontopathogens and the production of *IL-8* by comparing outcomes among patients carrying different haplotypes^21^,^22^,^23^. Despite the importance of *IL-8* in disease susceptibility, the biological explanation of the specific role of *IL-8* haplotype in the regulation of gene expression, and whether the ability and the activity of neutrophils modulated by the carriage of the *IL-8* haplotype have not been addressed previously. Hence, to determine the role of different *IL-8* haplotypes in transcriptional and translational activity of *IL-8* gene and to test whether or not it affects neutrophil migration, we used clustered regularly interspaced short palindromic repeats (CRISPR) RNA-guided Cas9 nucleases to edit *IL-8* gene with ease, speed and accuracy that permitted us to study functional aspects of *IL-8* haplotypes. The CRISPR/Cas9 system has been recently used in engineering the genomes^24^,^25^,^26^. This study represents a novel means to perform genome editing by using a naked double stranded DNA as a homologous repair template to discover the functional role of genes and in this report we focus on the functional effects related to the *IL-8* haplotypes.

## Results

### Cloning and validation of CRISPR/Cas9 for IL-8 genome-editing

**Figure 1:**
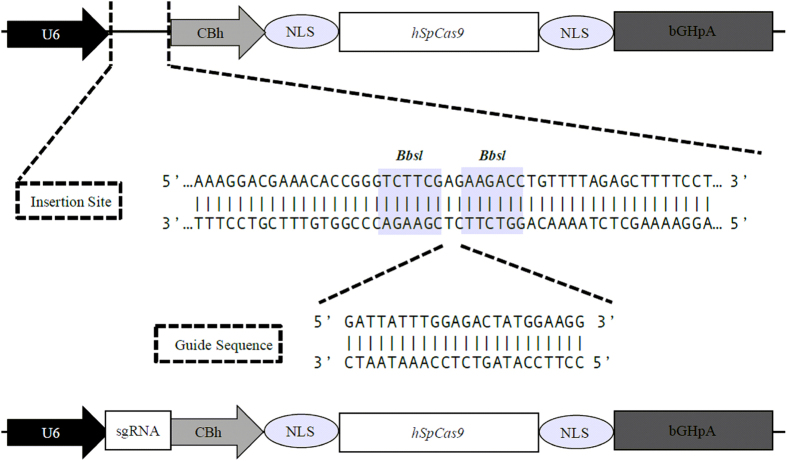
Schematic diagram of pX330-based vector with U6 promoter driving sgRNA and expression of Cas9 (hSpCas9). The expanded view illustrates the nucleotide sequence of *IL-8* spanning a portion of the guide sequence insertion site with *Bbsl* sites indicated for protospacer “guide sequence” (sgRNA) insertion.

**Figure 2:**
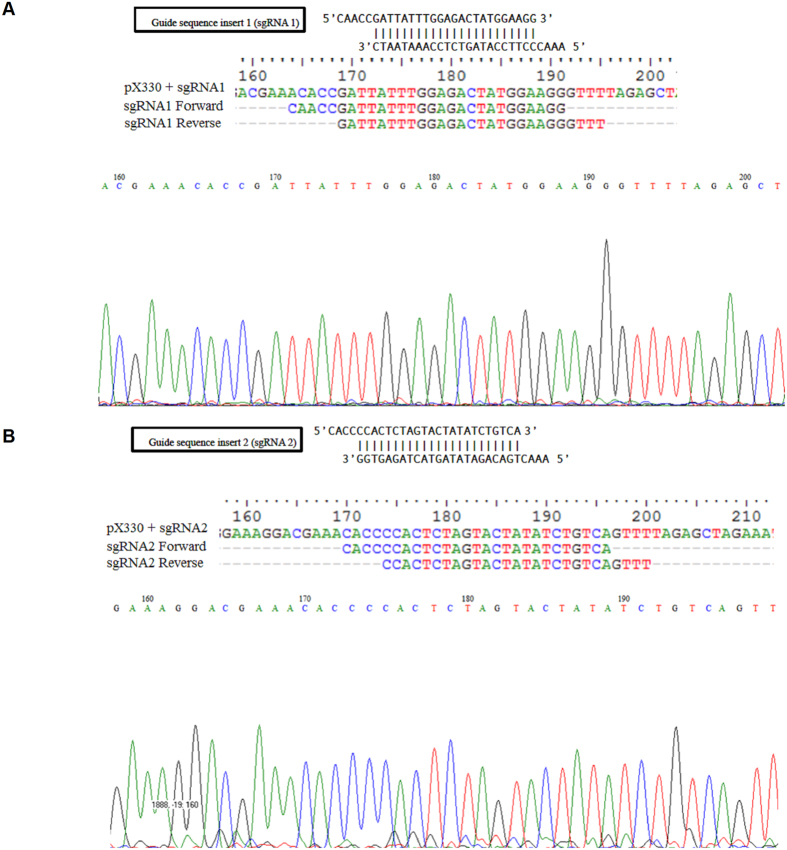
Sequence validations of insert targeting sequence of sgRNA1 **(A)** and sgRNA2 **(B)** with upstream *U6* promoter sequence on pX330 vector. The inserts were confirmed by sequencing both forward and reverse strands.

In order to test if the RNA-guided CRISPR/Cas9 system could be applied for gene double nicking, the single stranded sequences of the sgRNA1 and sgRNA2 were cloned into pX330 vector as described in methods section (Fig. 1). The constructs were verified by DNA sequencing using U6 promoter DNA sequencing primer (5′-CAAGGCTGTTAGAGAGATAATTGGA-3′). The DNA sequencing and multiple alignment of the region with sense and antisense single stranded oligos showed upstream *U6* promoter sequence and the inserted targeting sequence of sgRNA1 as well as sgRNA2 on pX330 vector (Fig. 2A,B).

### Generation of HEK293T cell lines carrying IL-8 haplotypes

**Figure 3:**
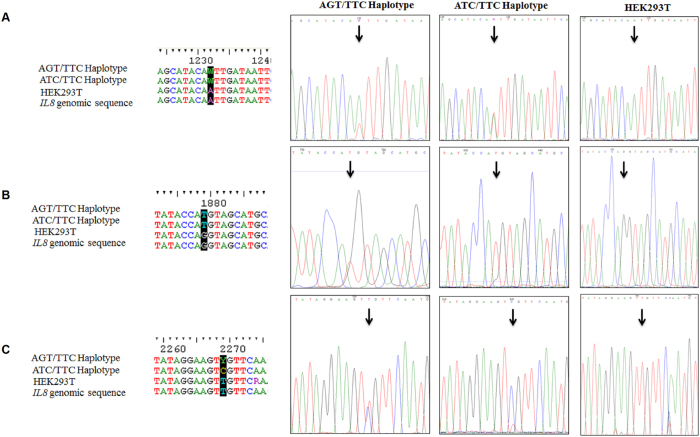
Generation and sequence validation of three cell lines. (**A**) HEK293T cells with ATT/TTC *IL-8* Haplotype; (**B**) HEK293T cells with ATC/TTC *IL-8* Haplotype; (**C**) HEK293T cells without *IL-8* Haplotype (wild type). Genomic PCR (gPCR) products were amplified from HEK293T cells transfected with pX330-sgRNA plasmids in addition to homologous repair templates that present different *IL-8* haplotypes (Arrows and dark background on the chromatogram and sequences showing corresponding nucleotides, respectively).

**Figure 4:**
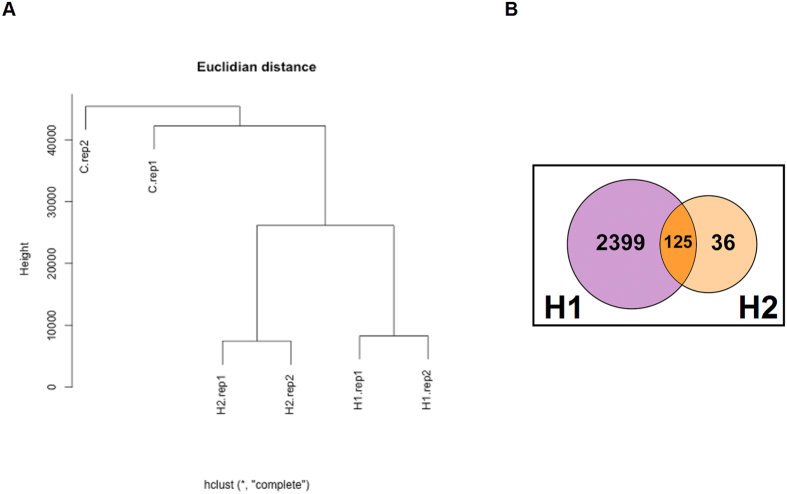
RNA-seq analysis of CRISPR/Cas9 edited HEK293T cells. RNA-seq was performed on duplicate samples (two independent experiments) to determine differential transcript expression of genome edited HEK293T cells. (**A)** Shows hierarchical clustering with Euclidian distance (Untreated: empty vector, H1: Haplotype 1 and H2: Haplotype 2. (**B)** Venn diagram showing number of transcripts that are significantly different in H1 and H2 compared to wild type cells.

HEK293T cells were transfected with the pX330-sgRNA1 and 2 plasmids in addition to homologous repair templates with different *IL-8* haplotypes separately. Positive clones were selected as described in the methods section. Results showed the generation of three conditions of edited and non-edited cell lines (Fig. 3): 1) AT genotype in the first SNP - rs4073 (alias -251), TT genotype in the second SNP - rs2227307 (alias +396), TC genotype at the third SNP - rs2227306 (alias +781); 2) AT genotype at the first SNP, TT genotype at the second SNP, CC genotype at the third SNP; 3) AA genotype at 1231 nucleotide position, GG genotype in 1877 nucleotide position, TT genotype in 2262 nucleotide position. CRISPR/cas9 method allowed us to create cell lines carrying *IL-8* haplotypes. Recent investigations suggest that there are off-target effects of cas9 nuclease creating undesired mutations^27^,^28^,^29^,^30^,^31^. Hence we further investigated the off-target effect of haplotype sgRNA in our genome edited HEK293T cells using RNA-seq analysis. We claim that the transcriptomes of ATC/TTC and ATT/TTC are similar to controls with respect to immunological pathways. To test this hypothesis we generated RNA sequencing data, two replicates each of ATC/TTC, ATT/TTC and control. With two replicates per condition we are limited in differential expression analysis with precision; hence we performed an informative enrichment analysis in the following way. We compared permuted data where we combine one ATC/TTC haplotype and one control into one group and the other ATC/TTC haplotype and the other control into a second group. We then compared the spectrum of T-Statistics in this permuted data, averaged over all possible permutations, to that from the unpermuted data, to obtain a putative set of 986 genes enriched for the differentially expressed genes at the False Discovery Rate (FDR) level of 0.5. Therefore we expect 50% of the genes on the list to be differentially expressed. As these will be a random sample of all differentially expressed genes, if any pathways are enriched among the differentially expressed genes then they will also be enriched in the putative set and as such will produce significant *p*-values in an enrichment analysis. If immunological genes were affected differently between haplotypes and control then this enriched list should display significant pathway enrichment for immunological pathway related genes. We ran the list through Ingenuity Pathway Analysis (IPA) producing nine significantly enriched pathways, the most significant of which has enrichment *p*-value of 6.89E-05. The significantly enriched pathways are: Remodeling of Epithelial Adherens Junctions, Mismatch Repair, ILK Signaling, Hereditary Breast Cancer Signaling, Epithelial Adherens Junction Signaling, EIFT Signaling, Glycolysis I, Polyamine Regulation in Colon Cancer and Androgen Signaling, none of which are related to immunological pathways. We similarly investigated ATT/TTC haplotype producing a list of 217 genes at the 0.5 FDR level. In this case the significantly enriched pathways were EIF2 Signaling, Polyamine Regulation in Colon Cancer and Pyruvate Fermentation. There were no immunological pathways enriched for ATT/TTC haplotype. Heirarchichal clustering with Euclidian distance od differentially expressed genes showed close similarities between haplotypes (Fig. 4A). We generated Venn diagram representing number of genes that are signficantly different between the haplotypes and control (Fig. 4B).

### ATC/TTC haplotype up-regulate IL-8 gene at transcriptional and translational level

**Figure 5:**
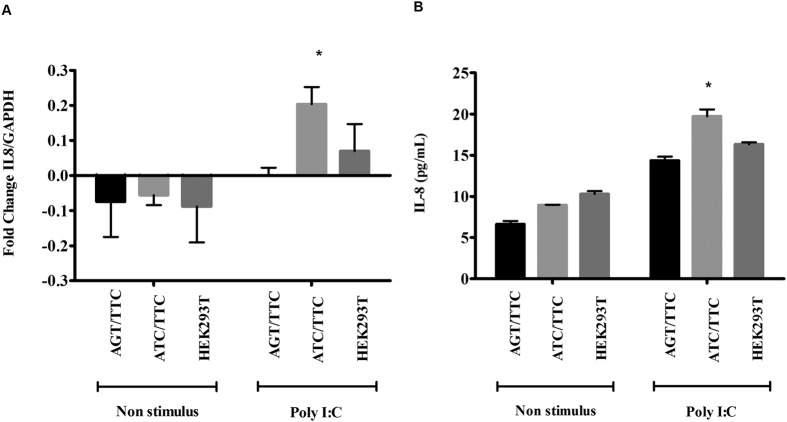
(**A)** *IL-8* mRNA levels in CRISPR/Cas9 edited HEK293T cells. Quantitative real-time PCR showing *IL-8* mRNA expression in edited and wild type HEK293T cells challenged with Poly I:C for 24 h. The un-stimulated cells served as negative control. ATC/TTC haplotype significantly up-regulated *IL-8* mRNA levels upon Poly I:C treatment. Values represent mean ± SD of at least three independent experiments (*p-value < 0.05). (**B)** *IL-8* protein expression in CRISPR/Cas9 edited HEK293T cells. ELISA was performed from edited and wild type cells stimulated with Poly I:C for 24 h. Un-stimulated cells served as negative control. ATC/TTC haplotype significantly up-regulated *IL-8* protein levels upon Poly I:C treatment. Values represent the mean ± SD of at least three independent experiments (*p-value < 0.05).

To identify the regulation of cytokines in gene edited and wild type cells, we stimulated with stimulation with a TLR3 agonist poly I:C to determine whether or not carrying the haplotype affects the transcriptional levels. After 24 h of stimulation, *IL-8* messenger RNA (mRNA) levels were measured by quantitative real-time PCR. The analysis revealed that Poly I:C stimulus enhanced *IL-8* mRNA expression compared to unstimulated negative control as expected (Fig. 5A). Interestingly, *IL-8* mRNA levels induced by cells carrying ATC/TTC haplotype was significantly higher when compared with *IL-8* mRNA levels induced by ATT/TTC haplotype under poly I:C stimulus (Fig. 5A).

The increase in mRNA levels in the cells prompted us to test the *IL-8* protein levels in the supernatants. Gene edited and wild type cells were stimulated with Poly I:C for 24 h as described above. Secreted *IL-8* was measured in the supernatant by ELISA. As expected poly I:C stimulated samples had higher levels of *IL-8* compared with un-stimulated samples (Fig. 5B). Similar to mRNA levels, *IL-8* protein levels induced by ATC/TTC haplotype was significantly higher when compared with the *IL-8* protein levels induced by ATT/TTC haplotype upon poly I:C stimulation (Fig. 5B).

### ATC/TTC IL-8 haplotype increase PMN transmigration

**Figure 6:**
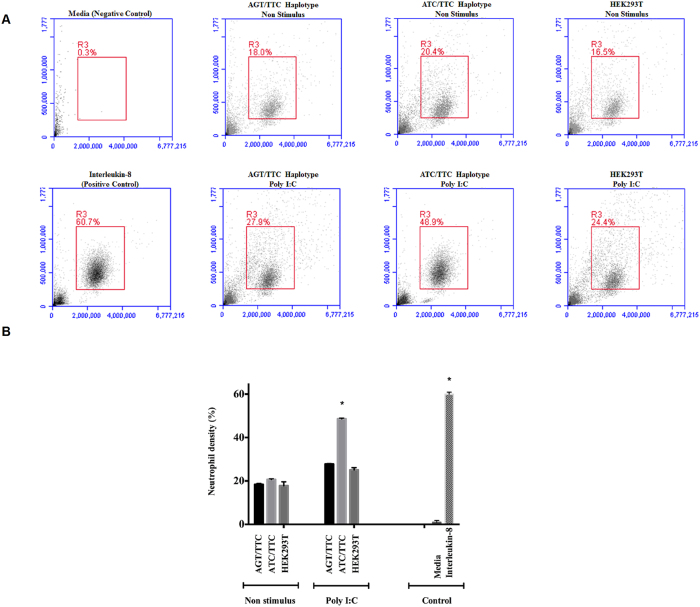
Trans-well assay for neutrophil transmigration. Genome edited and wild type cells were stimulated with Poly I:C and supernatant was used in transwell system to monitor whether or not cells are altered in their ability to promote PMN transmigration. After the migration assay, the average number of migrated PMN cells are analyzed by FACS **(A)** and plotted in a bar diagram where the results are expressed as the means ± SD obtained from 6 fields/group herewith negative and positive control **(B)**. Supernatant from ATT/TTC haplotype recruited significantly higher neutrophils compared to that of wild type. Statistical comparisons are from three independent experiments (*p-value < 0.05).

To test the functionality of different haplotypes on *IL-8* gene, we screened edited and wild type cells to determine whether any of the haplotypes are altered in their ability to promote PMN transmigration. As expected, the capacity to induce PMN transmigration in un-stimulated cells’ supernatant was lower than poly I:C stimulated cells. The response to negative control (DMEM media) was minimal contrary to the recombinant human *IL-8*, which is used as positive control, was the highest when compared to other conditions. It was also observed that the cells carrying ATC/TTC haplotype dramatically increased its capacity to induce PMN transmigration when compared with ATT/TTC haplotype with poly I:C stimulation (Fig. 6). This increase in migration could be concentration dependent as ATC/TTC cell type induced higher *IL-8* induction.

## Discussion

Interleukin-8 (*IL-8*) is considered an important chemokine in periodontal disease. This cytokine is produced by a variety of cells and may function in concert with other members of the cytokine family to regulate the host’s innate responses^2^,^32^,^33^,^34^. Specifically, this cytokine attracts leukocytes from the periphery to the sites of infection and activates them to become phagocytes. Intra-cutaneous administration of *IL-8 in vivo* induced local exudation and long lasting accumulation of neutrophils^35^. Although, there are other chemokines involved in neutrophil recruitment to the site of infection, *IL-8* receptor knock-out mice showed delayed neutrophil influx into the kidneys and bladder and were unable to eliminate bacteria from the tissues^36^,^37^. This suggests that *IL-8* is indispensable for neutrophil migration and function. Neutrophil function is not only important in acute infections but also plays a major role in chronic inflammatory disorders such as periodontitis, atherosclerosis, psoriasis, rheumatoid arthritis, inflammatory bowel disease, diabetes and cancer^38^,^39^.

Recent meta-analyses showed a positive association of -251(T/A) polymorphism on *IL-8* gene to chronic periodontitis^40^,^41^. Few case-control studies have investigated different haplotypes in the *IL-8* gene that were found to be associated with periodontitis^20^,^42^,^43^, such as the ATC/TTC haplotype whose carriers of this particular haplotype had two times higher disease susceptibility^20^. In spite of this, the *IL-8* protein levels in the GCF of patients were not correlated to the carriage of ATC/TTC haplotype^22^. This absence of correlation could be attributed to the limited sample size in that study, besides the individuals with chronic periodontitis enrolled in that study were not affected by the severe and generalized disease forms.

Faced with the lack of functional assays, we hypothesized that an *in vitro* study with more controlled conditions could be able to detect the potential influence of different haplotypes in the *IL-8* mRNA and protein levels. This present study demonstrated that the presence of ATC/TTC haplotype can up-regulate the *IL-8* in both mRNA and protein levels. These higher *IL-8* mRNA and protein levels coupled with neutrophil migration could explain the lower periodontopathogens levels found in patients carrying the ATC/TTC haplotype^21^,^44^. Also, high levels of *IL-8* may increase the inflammatory and immune response and subsequent damage to the integrity of the periodontium. We think that the present findings could explain why the periodontal destruction may occur in patients who were considered to be genetically susceptible to chronic periodontitis with a lower microbial challenge because of the presence of the *IL-8* ATC/TTC haplotype than in patients without it^21^,^44^.

The same ATC/TTC haplotype was also shown to be associated with bronchial asthma, while the rs4073T > A SNP when tested alone did not show significant association^45^. Other studies reported similar lack of association with chronic periodontitis when a SNP was analyzed individually, but when the haplotype was considered in the analysis, the association with the disease was revealed^43^,^46^. Therefore, these studies corroborate the idea that haplotypes are more powerful for detection of disease association than individual polymorphisms and they may give more information on the basis of disease^43^,^47^. Despite the previously reported functional role of rs4073T > A SNP^13^, a study by Hacking *et al.*^48^ failed to confirm this association^48^. Hacking *et al.*, and others suggested that the existence of another SNP (closer to rs4073T > A) could play a role in modulating *IL-8* gene expression^20^,^48^,^49^,^50^. Hence, we noticed in our study that the +781(C/T) SNP rs2227306 in the *IL-8* gene seems to influence the *IL-8* at both mRNA and protein levels, considering that the difference in the haplotypes analyzed here was related to the alleles in that position.

A study by Ahn *et al.*, in patients with idiopathic pulmonary fibrosis (IPF) showed increased *IL-8* levels in patients carrying the A allele at the rs4073T > A SNP^51^. The authors used a luciferase assay to measure the activity and determined the level of *IL-8* in the presence or absence of promoter SNP. They found increased luciferase activity in the presence of rs4073T > A SNP on the promoter of *IL-8* gene. The authors concluded that *IL-8* promoter SNP may increase susceptibility to the development of IPF via the up-regulation of *IL-8*^51^. By using similar reporter vector system, Meade *et al.*, investigated bovine *IL-8* promoter haplotypes *in vitro*. The authors found that the luciferase promoter carrying one of the haplotype *IL-8*-h2 (C–GTAC) highly up-regulated luciferase activity upon LPS and TNF stimulation confirming SNP functionality and suggesting a differential transcriptional factor binding to *IL-8*-h2 promoter (such as C/EBP, Oct-1, NFκB and NFAT)^52^. Hacking *et al.*^48^ observed that C/EBPb (CCAAT/enhancer binding protein-beta) bound to the transcriptional complex in the presence of rs4073T > A allele in respiratory epithelial cells but not in primary lymphocyte cells suggesting cell type specificity in transcriptional regulation^48^. Although, the luciferase reporter assays is an accepted technique that can be used to test the functionality of SNP, we believe that it is an artificial system that does not contain complex regulatory elements found on the chromatin. To overcome this gap in the functional analysis of haplotypes and test our hypothesis, we adopted a novel technique called clustered regularly interspaced short palindromic repeats (CRISPR) RNA-guided Cas9 nuclease system^53^ to edit the *IL-8* gene in human embryonic kidney cell line (HEK293T). This technology is currently a burning topic in the field of genome editing and an invaluable tool to engineer genomes of choice^54^. For generating *IL-8* editing cell lines, we choose HEK293T cells as the model system as this cell line is commonly used with CRISPR/Cas9 technology, making them a well-established model system to test the efficacy of RNA-guided endonucleases in gene editing^55^,^56^. With this technique, we were able to successfully edit the *IL-8* gene within the HEK293T genome to carry different haplotypes with unprecedented precision and ease. Further, we were able to test the effect of different haplotypes in the *IL-8* gene transcription, protein and also functionally evaluate their ability in modulating neutrophil transmigration. The methodology used for this purpose offers a simple and applicable framework for generating validated edited cell lines. Our workflow is adapted from previously published methods and/or in house developed protocols especially utilizing naked double stranded DNA for genome editing that can be divided into four phases: 1) sgRNA target design^30^; 2) sgRNA expression vector construction^57^; 3) Homologous repair templates to homology-directed repair (HDR); 4) Cell culture and transfections. This methodology using the CRISR/Cas9 system demonstrated that the presence of ATC/TTC haplotype can up-regulate the *IL-8* in HEK293T cells upon Poly I:C stimulation. Roy *et al.*, generated two clonal cell lines targeting exon 1 of the *WNK1* gene by using a similar technique within a short period of time^55^. Since the CRISPR/Cas9 system is still new and being validated, we found fewer publications related to SNP and haplotype editing in general. Although, there is tremendous development and usage of this technique within the last two years, unfortunately, programmable nucleases such as zinc-finger nucleases, transcription effector nucleases, RNA-guided engineered nucleases (RGENs) and the CRISPR/Cas9 system can induce off-target mutations within the genome^27^,^28^,^29^,^30^,^31^. Various technological platforms are still being developed to address off-target effects of these nucleases^58^,^59^ to be able to modify or alter sgRNA sequences to minimize or eliminate undesired cleavage by guided nucleases. In this regards, we used genome wide RNA-seq analysis to determine the differential expression of transcripts in our genome edited cells. Although, we found transcripts that were differentially expressed in ATC/TTC haplotype compared to control, but this accounted for ~2.8% of transcriptome difference (986 out of 34,917 transcripts). However, we found no enrichment for immunological pathways in genome edited cells. Nevertheless, we are in agreement that one must be extremely careful in designing sgRNA to alter genome of interest.

In summary, our data demonstrate that the ATC/TTC in the *IL-8* gene can have a positive outcome on the transcriptional and translational levels of *IL-8* gene and thus may modulate neutrophil recruitment at the site of infection. Taken together, because of the critical role neutrophils have in periodontal disease, it is plausible that the carriage of a particular *IL-8* haplotype ATC/TTC may contribute to periodontal disease susceptibility.

## Material and Methods

### CRISPR-Cas9 vector construction

The bicistronic pX330 vector was procured from Addgene (pX330-U6-Chimeric_BB-CBh-hSpCas9 was a gift from Feng Zhang (Addgene plasmid #42230)). This vector constructed with cDNAs encoding human codon-optimized *Streptococcus pyogenes* Cas9 (hSpCas9) that binds to and cleaves DNA, and an adaptable CRISPR RNA (crRNA)/trans-activating crRNA chimera containing adjacent *BbsI* cloning sites for protospacer “guide sequence” (sgRNA) insertion (Fig. 1). The plasmid was transformed into DH5α competent cells (Life Technologies, US) and were selected on 100 μg/mL Ampicillin (Sigma-Aldrich, Saint Louis, MO) LB plates. A single +ve bacterial colony was inoculated in 1mL of LB medium and allowed to grow on a shaker at 37 °C. After overnight culture, plasmid was isolated by using a PureLink Quick Plasmid Miniprep kit (Invitrogen, Carlsbad, CA).

### sgRNA Target Design

In order to improve the specificity of off-targets, Target Finder program from Feng Zhang lab was used to design specific sgRNA^30^. DNA cleavage requires synergistic interaction of two independent specificity-encoding DNA-binding modules. This allowed us to define parameters for the selection of sgRNA pairs that facilitate effective double nicking in which gRNA1 had to be downstream to the first SNP (-251) as well as gRNA2 upstream to the third SNP (+781) of the *IL-8* gene. Therefore 28-bp and 27-bp guide sequences targeting DNA were selected based on predicted high specificity protospacer adjacent to motif (PAM) target sites on the *IL-8* gene. Two complementary oligos containing guide sequence including *BbsI* ligation adapters were synthesized by IdtDNA technologies for each sequence.

### sgRNA expression vector construction

The sense and antisense single stranded DNA of each oligos pair (100 mM) were annealed using 0.5 μL of T4 polynucleotide kinase (New England Biolabs, MA) and 1 μL 10X T4 Ligation Buffer in a total volume of 10 μL by incubating the oligo mix at 37 °C for 30 min, then 95 °C for 5 min, followed by a ramp to 25 °C at 5 °C/min^60^. The annealed oligos (gRNA1 and gRNA2) were ligated into the *BbsI*-digested pX330 vector using 2 μL 10x Fast Digest Buffer (Life Technologies, Paisley, UK), and 0.5 μL of T7 DNA ligase (New England Biolabs, Ipswich, MA)^57^. The ligation mixture was treated with Plasmid Safe exonuclease and transformed in One-Shot chemically competent DH5α cells (Life Technologies, Paisley, UK). Transformed clones were selected on 100 μg/mL Ampicillin (Sigma-Aldrich, Saint Louis, MO) LB plates. After plasmid DNA extraction (Qiagen, CA), the sequence of the construct was verified by automated DNA sequence analysis performed at the University of Pennsylvania’s DNA sequencing core facility.

### Homologous repair template

Generation of a targeted double-strand DNA break (DSB) creates multiple repair choices^53^. A DSB may be repaired via homology-directed repair (HDR) by using homologous DNA as a template. For our experimental purpose, homologous repair templates were constructed in two steps. First, we sequenced DNA of de-identified human subjects from previous study^20^ in order to confirm and select DNA sample that represented ATT/TTC haplotype, and one sample that represented ATC/TTC haplotype. Second, we PCR-amplified the fragment corresponding to the same region which was excised by complex CRISPR/Cas9 with guide sequence insert 1 and guide sequence insert 2 (Table 1). The products of the reactions were verified on 1% agarose gel. The fragments of interest were excised from the gel for purification using PureLink purification kit (Invitrogen, Carlsbad, CA) following the manufacturer’s instructions and verified by DNA sequencing.

### Cell Culture and Transfection

HEK293T cells were cultured in six-well plates to 50–60% confluence with pre-warmed starving medium (DMEM supplemented with 10% dialyzed fetal bovine serum and 1% penicillin/streptomycin) and incubated in a 5% CO_2_ incubator at 37 °C. Transfections were performed in four different conditions: 1. Transfection with two specific CRISPR/Cas9 plasmid constructs in addition to homologous repair template with ATT/TTC haplotype; 2. Transfection with two specifics CRISPR/Cas9 constructs in addition to homologous repair template containing ATC/TTC haplotype; 3. Transfection with two specifics CRISPR/Cas9 constructs; 4. Negative control (Cells transfected with TE (1X) and transfection reagent. A total of 2.5 × 10^5 ^cells were transfected with 0.5 μg of each sgRNA plasmid and 0.2 μg of DNA template using the GenMute Reagent (SignaGen Laboratories, MD) according to the manufacturer’s instructions. The medium was replaced with fresh medium the following day and at 48 h post-transfection. Seventy-two hours post-transfection, the cells were harvested, low density of cells counted, diluted to 1 × 10^4 ^cells/ml and 2 μl/well was transferred to 96 well plates for clonal selection. The genomic DNA was extracted using QIAmp DNA Mini Kit (Qiagen, CA). A PCR reaction was performed using guide sequence insert 1 forward and guide sequence insert 2 reverse primers (Table 1). The homologous repair template inserts with the SNPs were confirmed by DNA sequence analysis performed at the University of Pennsylvania’s DNA sequencing core facility. Out of 10 clones each, 4 clones from ATT/TTC haplotype and 1 clone from ATC/TTC haplotype were found to be positive.

### RNA-seq analysis

Raw sequence reads obtained from Illumina HiSeq 2500 V4 100bp single-read sequencing were filtered to retain only high quality reads. Data were aligned to the genome with STAR^61^. The low level processing was performed with the PORT pipeline (github.com/itmat/Normalization). PORT removes ribosomal RNA by BLAST alignment to reference rRNA. PORT then equalizes exon signal across all samples by random sampling of reads to produce SAM files with an equal number of exon mappers in all samples. PORT normalizes for several other factors including highly expressed highly variable elements. Data were then quantified at the gene level using ENSEMBL gene annotation. Differentially expressed genes between control and CRISPR/cas9 derived haplotype samples were determined using FDR control by PaGE^62^. Pathway analysis was performed by Ingenuity IPA (www.ingenuity.com/products/ipa). All pathways with *p*-value below 1.0E-4 were considered significant, which in practice in pathway analysis is a liberal criteria. A liberal criteria was required for the argument that no immunological pathways were affected.

### Stimulation of HEK293T cells

TLR3 pathway induces the *IL-8* expression when stimulated by poly I:C, a TLR3 agonist^63^. To perform the functional analysis, HEK293T cells that were genome edited to carry haplotypes and along with control cells were cultured in six-well dishes to 70–80% confluence with pre-warmed starving medium (DMEM supplemented with 10% dialyzed fetal bovine serum and 1% penicillin/streptomycin,) and incubated in fresh starving medium in a 5% CO_2_ incubator at 37 °C. A total of 0.5 × 10^6 ^cells were stimulated with Poly I:C of 5 μg/mL for the 24 h. There were six conditions: 1) HEK293T cells with ATT/TTC haplotype stimulated with TLR3 agonist poly I:C; 2) HEK293T cells with ATC/TTC haplotype stimulated with TLR3 agonist poly I:C; 3) HEK293T cells stimulated with TLR3 agonist poly I:C; 4) HEK293T cells with ATT/TTC haplotype non-stimulated; 5) HEK293T cells with ATC/TTC haplotype non-stimulated; 6) HEK293T cells non-stimulated. Subsequently, experiments were carried out for the assessment of *IL-8* gene expression, quantitation of *IL-8* protein and Neutrophil migration.

### Neutrophil isolation

Five milliliter of whole blood was obtained by venipuncture into tubes containing sodium heparin (BD Biosciences, CA). Neutrophils were isolated according to Nauseef^64^. Neutrophils were washed several times, and re-suspended in pre-warmed DMEM medium. Human neutrophils were isolated from healthy volunteers with signed informed consent approved by the University of Pennsylvania’s Institutional Review Board (IRB). All experimental protocols were approved by the University of Pennsylvania’s IRB. The investigation was conducted in accordance with the principals of the Declaration of Helsinki.

### PMN transmigration assay

We have adapted a neutrophil transmigration assay protocol as previously described^65^. The assay was conducted in a modified 24-well (3.0 μM pore size) chamber (Corning Incorporated, NLD). Eight hundred microliters of the supernatant described in step above was placed in each well. The recombinant human *IL-8* (R&D Systems, Minneapolis, MN) was used as a positive control as well as only DMEM medium was used as negative control. Subsequently PMNs (1 × 10^6^) were added to the top (basolateral) chamber and incubated at 37 °C for 2 h. PMNs that migrated across the bottom (apical) chamber were quantified by fluorescence activated cell sorting (FACS) using BD Accuri™ C6 flow cytometer.

### Quantitative real-time RT-PCR

Total RNA of each condition was isolated using the RNeasy kit (Qiagen, CA) according to the manufacturer’s instructions. RNA integrity and quantity were checked by spectrometry with a NanoDrop ND1000 spectrophotometer. Five micrograms total RNA was used for cDNA synthesis with the High Capacity cDNA Archive kit (Applied Biosystems, CA). Real-time PCR was performed using the cDNA (50 ng) with *IL-8* as primer and probe and GAPDH as endogenous control on ABI 7500 Fast system (Applied Biosystems, CA) in the presence of TaqMan DNA polymerase according to Benakankakere *et al.*^66^. The data were analyzed by DDCT method^67^ normalizing mRNA level to mRNA AGT/TTC with poly I:C stimulus.

### ELISA

Two hundred microliters of culture supernatant described in step “Stimulation of HEK293T cells with Poly I:C” was used. *IL-8* level was measured by enzyme-linked immunosorbent assay (ELISA) using a commercially available kit (BD Biosciences, CA) according to the manufacturer’s instruction. The absorbance was read at 450 nm. The concentrations were expressed in pg/ml.

### Statistical Analysis

Data displayed for each figure is from at least three independent experiments with a mean (standard deviation) of at least three independent data points/condition. Each experiment has been repeated multiple times yielding similar results. Statistical analysis was done using GraphPad Prism 5.0 (San Diego, CA). Data were analyzed with one-way ANOVA followed by Tukey’s multiple comparison tests. Statistical differences were considered significant at the p < 0.05 level and indicated by an asterisk (p < 0.05 (*)).

## Additional Information

**How to cite this article**: Benakanakere, M. R. *et al.* Investigation of the functional role of human Interleukin-8 gene haplotypes by CRISPR/Cas9 mediated genome editing. *Sci. Rep.* **6**, 31180; doi: 10.1038/srep31180 (2016).

## Figures and Tables

**Table 1:** Targeting sites chosen for Cas9-HR and oligonucleotides used to generate the corresponding gRNAs.

Guide sequence insert 1 Forward	5′CAACCGATTATTTGGAGACTATGGAAGG 3′
Guide sequence insert 1 Reverse	5′AAACCCTTCCATAGTCTCCAAATAATC 3′
Guide sequence insert 2 Forward	5′ CACCCCACTCTAGTACTATATCTGTCA 3′
Guide sequence insert 2 Reverse	5′AAACTGACAGATATAGTACTAGAG**TGG** 3′
